# Wide range temperature-dependent deformation and fracture mechanisms for 8701 under dynamic and static loading

**DOI:** 10.1039/c8ra01169a

**Published:** 2018-04-18

**Authors:** Hong-fu Guo, Yan-qing Wu, Feng-lei Huang

**Affiliations:** State Key Laboratory of Explosion Science and Technology, Beijing Institute of Technology Beijing China wuyqing@bit.edu.cn huangfl@bit.edu.cn

## Abstract

Although the RDX-based composite explosive 8701 explosive 8701 has been widely used to achieve military goals, its mechanical properties have not been carefully investigated. In the present study, we focused on the mechanical response of 8701 at a wide range temperature from −125 °C to 100 °C under both quasi-static (about 0.001 s^−1^) and high-rate compression loading (about 600 s^−1^). The stress–strain curves exhibit different tendencies at different temperatures for both quasi-static and high strain-rate loading. The failure stress and elastic/storage modulus present important temperature-dependence. Differential scanning calorimetry (DSC) tests showed that the glass transition temperature and softening temperature of 8701 are 11.61 °C and 15.14 °C respectively, which is lower than that of the binder (with glass transition temperature of 25 °C and softening temperature 38 °C). For the quasi-static loading, scanning electron microscopy (SEM) observations revealed that 8701 shows an interface debonding failure mode along the binder phase below 15 °C, while the mechanical behavior of 8701 is dominated by softening behavior of the binder above 38 °C. For high-rate loading, 8701 shows a mixture of interface debonding and trans-granular cleavage when below 15.14 °C.

## Abstract 2

The mechanical properties of 8701 were tested over a wide temperature range of −125 °C to 100 °C using the MTS Landmark hydraulic servo test system and SHPB.

## Graphical abstract

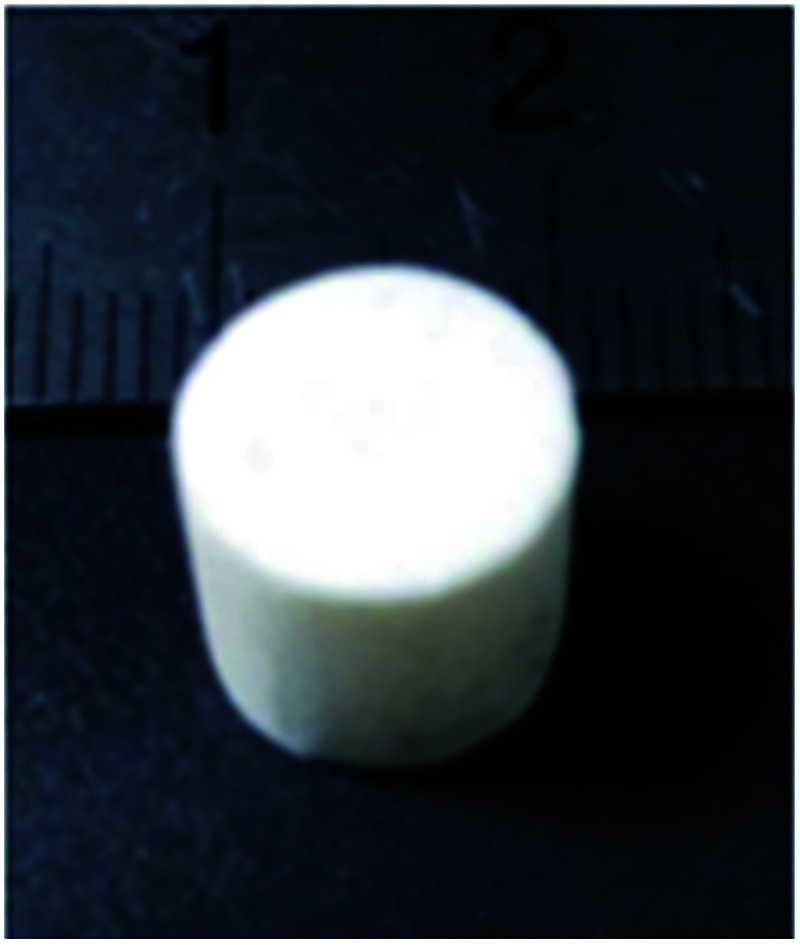

## 1. Introduction

Polymer bonded explosives (PBXs) are commonly used as energetic fillings in various weapon systems due to their low sensitivity and high detonation performance. The strain-rate and temperature strongly affect the mechanical responses of PBXs under compression and tension. Various experiments have been conducted on PBXs.^1–7^ Funk *et al.* tested the stress–strain response of PBX9501 at different temperatures and strain rates and found that the compressive strength decreases with increasing temperature and strain rate.^8^ Gray III *et al.* and Blumenthal *et al.* investigated the compression properties of PBX9501 and PBX9502 over a wide temperature range of −55 °C to +50 °C.^9,10^ Cady *et al.* studied three different formulations of explosive binders.^11^ Li *et al.* investigated the uniaxial compressive stress–strain behavior of three PBXs and found that the yield strength of PBXs is negatively correlated with temperature and positively correlated with strain rate.^12,13^ The compressive and tensile properties of PBX9501 and 9502 have been investigated over broad temperature ranges and drastically different loading strain rates.^14^ The dynamic response of an aluminized PBX over a temperature range of −55 °C to −2 °C and under a fixed loading strain rate has been tested using the split Hopkinson pressure bar (SHPB).^15^ Temperature strongly affects the mechanical response of PBXs. Few studies have investigated the properties of PBXs under dynamic and static loading over a wide temperature range. The mechanical properties of PBXs vary with different binders.^16,17^ 8701 was invented at the Beijing Institute of Technology. Its main components are cyclotrimethylene trinitramine (RDX) crystals and polyvinyl acetate. Given that polyvinyl acetate is easily affected by temperature change, the mechanical properties of 8701 are highly temperature-dependent. Nevertheless, the mechanical properties of the polyvinyl acetate binder and PBXs fabricated with polyvinyl acetate binders have been rarely discussed.

This study aims to identify the effects of a wide temperature range on the mechanical properties of 8701. 8701 was tested using the SHPB over a temperature range of −100 °C to 100 °C. Quasi-static compression data on the same materials were also obtained. To efficiently investigate the effects of temperature on 8701, similar experiments were performed on samples at a fixed strain-rate. The surface fracture patterns of PBX8701 specimens obtained at different temperatures were investigated using scanning electron microscopy (SEM).

## 2. Experimental investigation

### 2.1 Materials and preparation

**Fig. 1:**
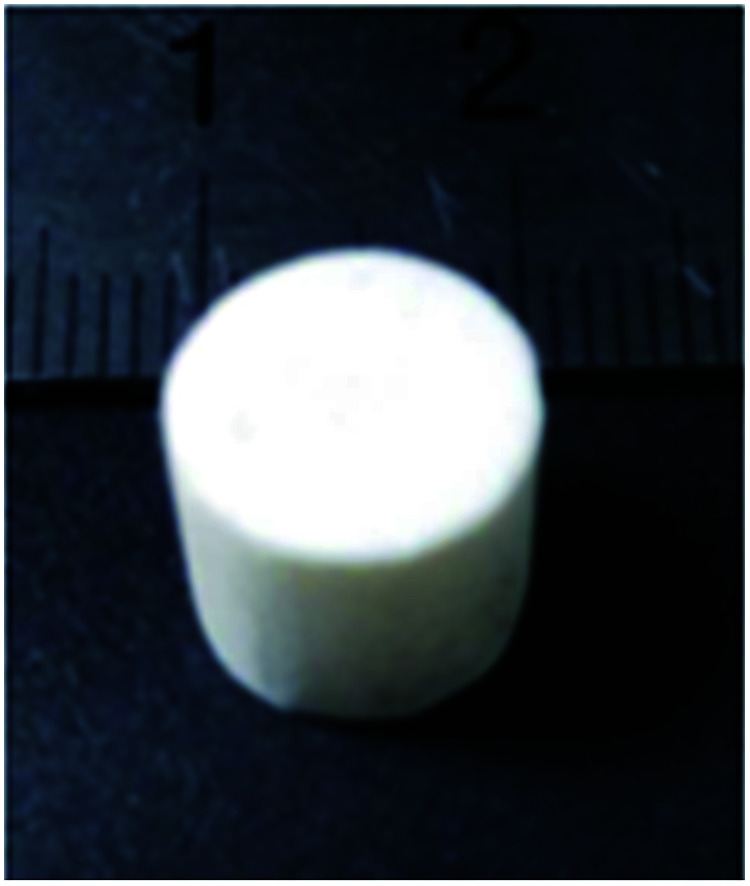
The specimen used for the quasi-static compression test.

**Fig. 2:**
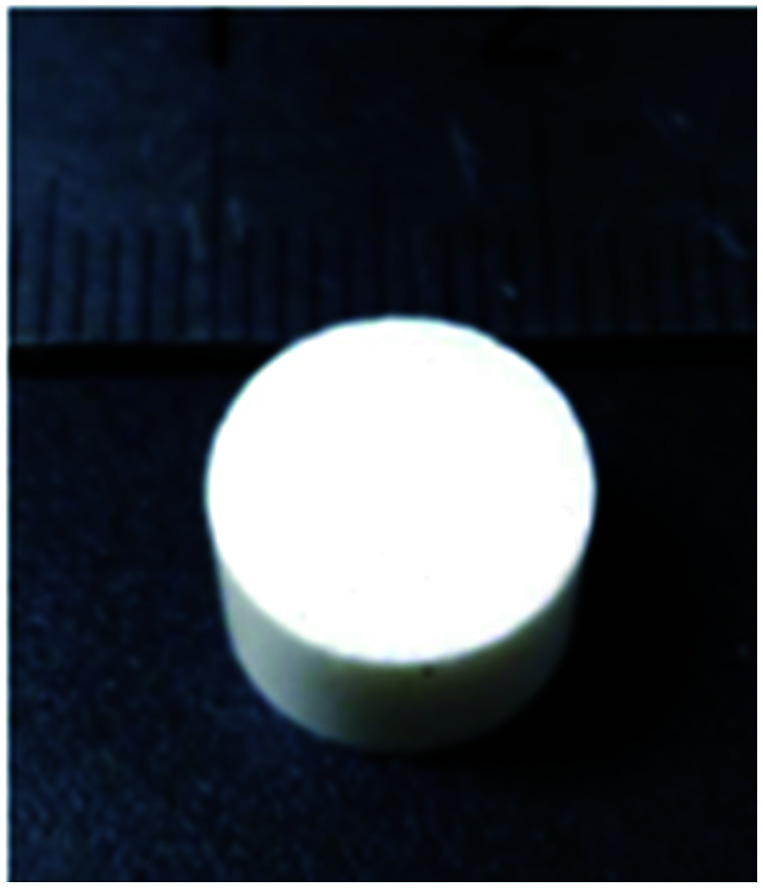
The specimen used for the SHPB test.

This investigation was performed on 8701, which is composed of 94.5 wt% RDX, 3 wt% dinitrotoluene, 3 wt% polyvinyl acetate and 0.5 wt% stearate. The 8701 samples used in this study were compression-molded using 8701 powder, which was prepared at the Beijing Institute of Technology. As shown in Fig. 1 and 2, the specimens used for the quasi-static and SHPB compression tests were machined to dimensions of ∼10 mm diameter × ∼10 mm and ∼10 mm diameter × ∼6 mm, respectively. The density of the samples was kept constant at 1.700 ± 0.001 g cm^−3^.

### 2.2 Quasi-static compression tests

**Fig. 3:**
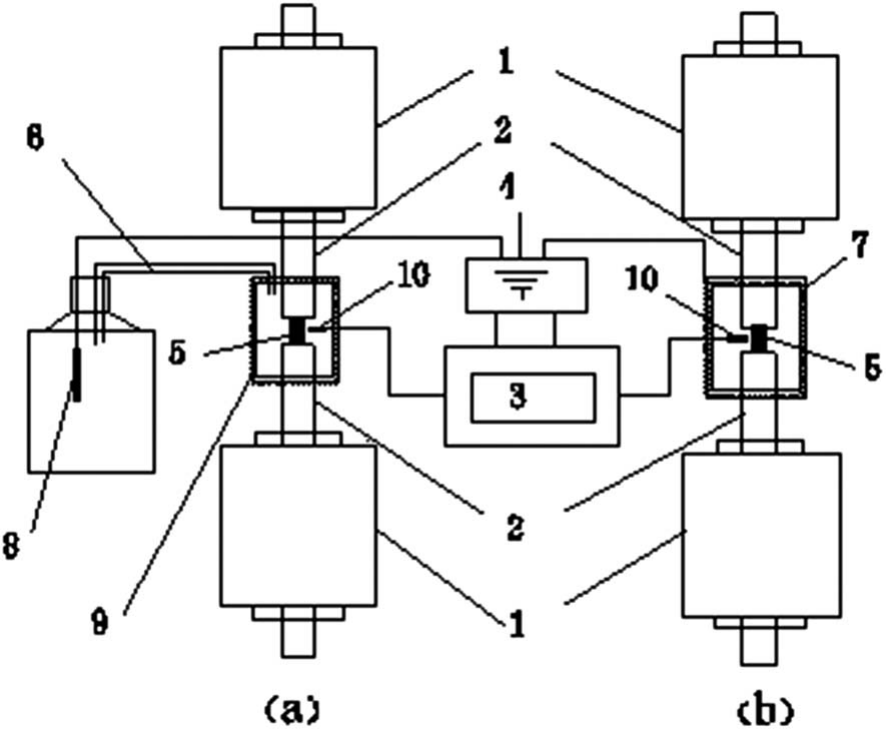
A schematic diagram of the quasi-static compression test at low (a) and high (b) temperatures: (1) hydraulic actuator (MTS), (2) pressing head (MTS), (3) temperature-control system, (4) electric source, (5) 8701, (6) air-ducts, (7) heating-ring, (8) electric heating wires, (9) heat preservation shell and (10) temperature sensor.

Quasi-static compression tests were conducted using an MTS Landmark hydraulic servo test system at the State Key Laboratory of Explosion Science and Technology, Beijing Institute of Technology. Quasi-static compression tests were conducted at a strain rate of 0.001 s^−1^ at different temperatures. The load and strain measurements were performed using an Instron load cell. A customized system was used to control the temperature changes. The samples were cooled using liquid nitrogen gas within a heat preservation shell held at the pressing heads of the MTS and heated using a heating ring. The specimen temperature was monitored using a temperature sensor. A temperature-control system was used to obtain the temperature measurements in real time when the specimen was loaded onto the MTS test system. A schematic diagram of the quasi-static compression test at low and high temperatures is shown in Fig. 3.

### 2.3 SHPB compression tests

**Fig. 4:**
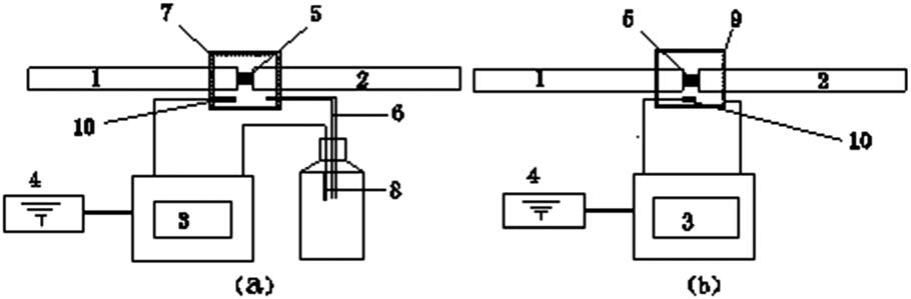
A schematic diagram of the SHPB compression test performed at low (a) and high (b) temperatures: (1) incident bar (SHPB), (2) reflection bar (SHPB), (3) temperature-control system, (4) electric source, (5) 8701, (6) air-ducts, (7) heating-ring, (8) electric heating wires, (9) heat preservation shell and (10) temperature sensor.

The 8701 samples were subjected to SHPB compression tests at the State Key Laboratory of Explosion Science and Technology, Beijing Institute of Technology. The SHPB was equipped with TC4 bars with a diameter of 14 mm. When compared with the maraging steel bars traditionally utilized in Hopkinson-Bar studies on metallic materials, TC4 bars provide better signal-to-noise levels, which is necessary for testing extremely low strength materials. The lengths of the bullet, incident bar, transmission bar, and striker bar were 20, 120, 120, and 40 cm, respectively. The incident and reflected strain were measured using electronic strain gauges on the incident bar, and the transmitted strain was measured using semiconductor strain gauges on the transmission bar. Dynamic tests were conducted at eight different temperatures and a fixed strain rate. The samples were subjected to a wide temperature range of −100 °C to 100 °C using a customized system developed at the State Key Laboratory of Explosion Science and Technology. In this system, the samples were cooled using liquid nitrogen gas within a heat preservation shell held at the heads of the Hopkinson bar and heated using a heating ring. The liquid nitrogen was heated using electric heating wires. The gas would enter the heat preservation shell containing the 8701 sample through air ducts. The specimen temperature was monitored using a temperature sensor (PT100) in the shell. The temperature-control system enabled the measurement of temperature in real time when the specimen was loaded between the bars. The temperature-control system had a precision of ±2 °C. A schematic diagram of the SHPB compression test performed at low and high temperatures is shown in Fig. 4.

## 3. Results and discussion

### 3.1 Quasi-static tests

**Fig. 5:**
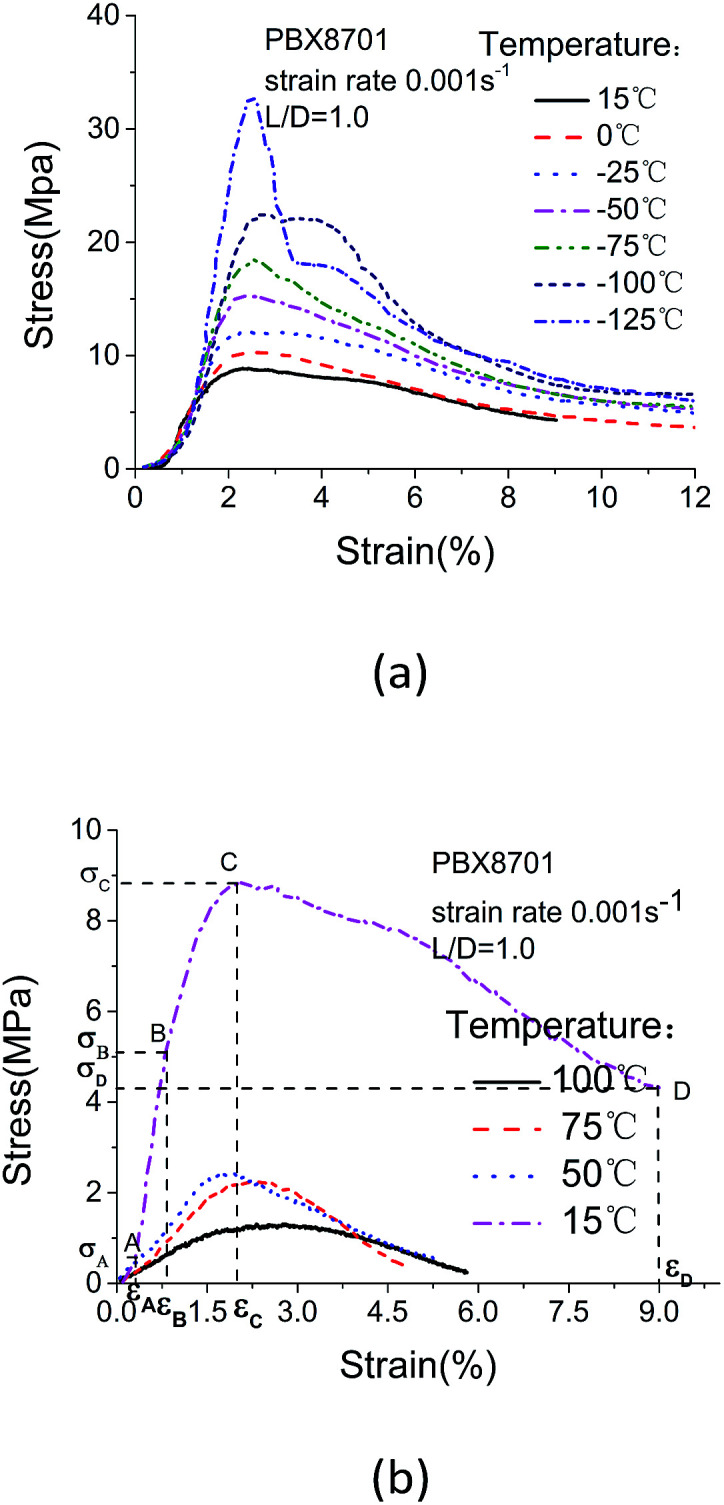
Stress–strain data obtained at (a) low and (b) high temperature.

The stress–strain curves of 8701 subjected to quasi-static tests at low and high temperatures are shown in Fig. 5(a) and (b), respectively.

The stress–strain curves demonstrate the strong temperature dependence of quasi-static compression. The fracture strain of 8701 is close to 2.5% at temperatures of less than 15 °C (Fig. 5(a)), and it then increases at temperatures of more than 50 °C (Fig. 5(b)). The development of the stress–strain curves of 8701 at room temperature is divided into four periods: 0–A, primary crack compression and closure of holes; A–B, linear elastic deformation; B–C, plastic yielding during which microcracks in the material expand rapidly and the material breaks at point C; C–D, material breakage, during which 8701 incompletely loses the ability to resist force. The residual strength of 8701 enables the material to continue bearing force and deformation. The compaction phase (0–A) is negligibly affected by low temperatures and is mainly affected by internal cracks and holes in the material. Temperature has little effect on the initial cracks and holes. The plastic yielding phase (B–C) continues to decrease, whereas the linear elastic phase (A–B) increases. At room temperature, the material yields and begins to flow under a strain of approximately 2.5%. Reducing the temperature to −50 °C slightly increases the strength, but does not affect the shape of the stress–strain curve; the strength, however, drastically increases at −75 °C and considerably increases further at −100 °C. 8701 becomes increasingly brittle as the temperature decreases and behaves in a fully brittle manner at −125 °C. As shown in Fig. 5(b), the stress–strain curves drastically change as the temperature increases from 15 °C to 50 °C. This change is related to binder softening. The softening temperature (*T*_s_) of polyvinyl acetate is 38 °C. The mechanical properties of 8701 are mainly determined by polyvinyl acetate and RDX organic crystals, but are considerably influenced by the binder at high temperatures.

**Fig. 6:**
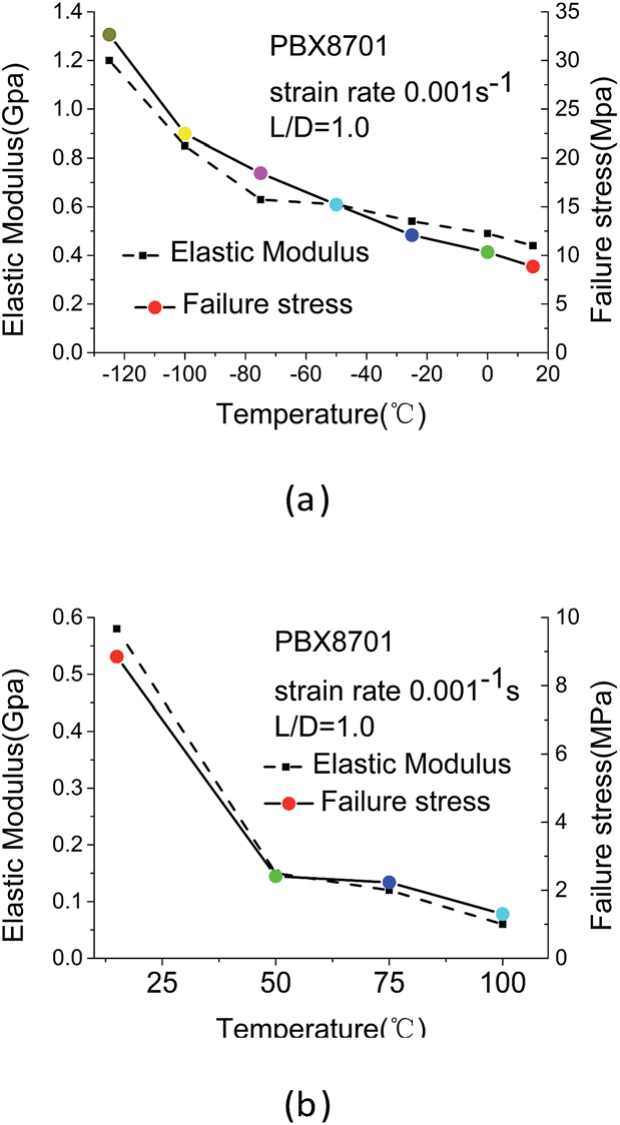
Failure stress and elastic modulus at low and high temperatures.

The failure stress and elastic modulus are negatively correlated with temperature, as shown in Fig. 6.

The curves show that 8701 increases in strength and becomes brittle with decreasing temperature. The failure stress of 8701 increases from 1.3 MPa at 100 °C to 32 MPa at −125 °C. This increase is accompanied by a 25-fold increase in the apparent loading modulus of the material. The development of failure stress and elastic modulus roughly consists of four overlapping stages: the viscoelastic stage (100–50 °C), the glass-transition and binder-softening stage (50–15 °C), the glass stage (15–75 °C), and the brittleness stage (−75 °C to 125 °C). During the viscoelastic stage, the binder has completely softened and even reached the viscous flow state. Thus, the failure stress and elastic modulus are low at this stage. Extreme changes occur during the glass-transition and softening stage of the binder, which account for the glass transition and softening of 8701. The glass-transition temperature (*T*_g_) and softening temperature (*T*_s_) of the binder are highly similar to those of 8701.^18,19^ The *T*_g_ of polyvinyl acetate is 25 °C (from public disclosure), whereas that of 8701 is 11.61 °C (from the DSC experiment). The *T*_s_ of the binder is 38 °C (from public disclosure), and that of 8701 is 15.14 °C (from the DSC experiment). The *T*_g_ and *T*_s_ of polyvinyl acetate and 8701 are highly similar. The glass transition and softening of polyvinyl acetate cause the glass transition and softening of 8701. During the glass stage, the failure stress and elastic modulus slowly increase with temperature. The failure stress and elastic modulus rapidly increase with temperature at temperatures of less than −75 °C. 8701 became a completely brittle material. By using a molecular dynamics method and compass force field, Zhu *et al.* found that the modulus of RDX organic crystals rapidly increases with decreasing temperature. Therefore, speculation that the contribution of RDX energetic crystals to the elastic modulus of 8701 exceeds that of the binder at the brittleness stage is reasonable.^20^

### 3.2 Dynamic compression tests

**Fig. 7:**
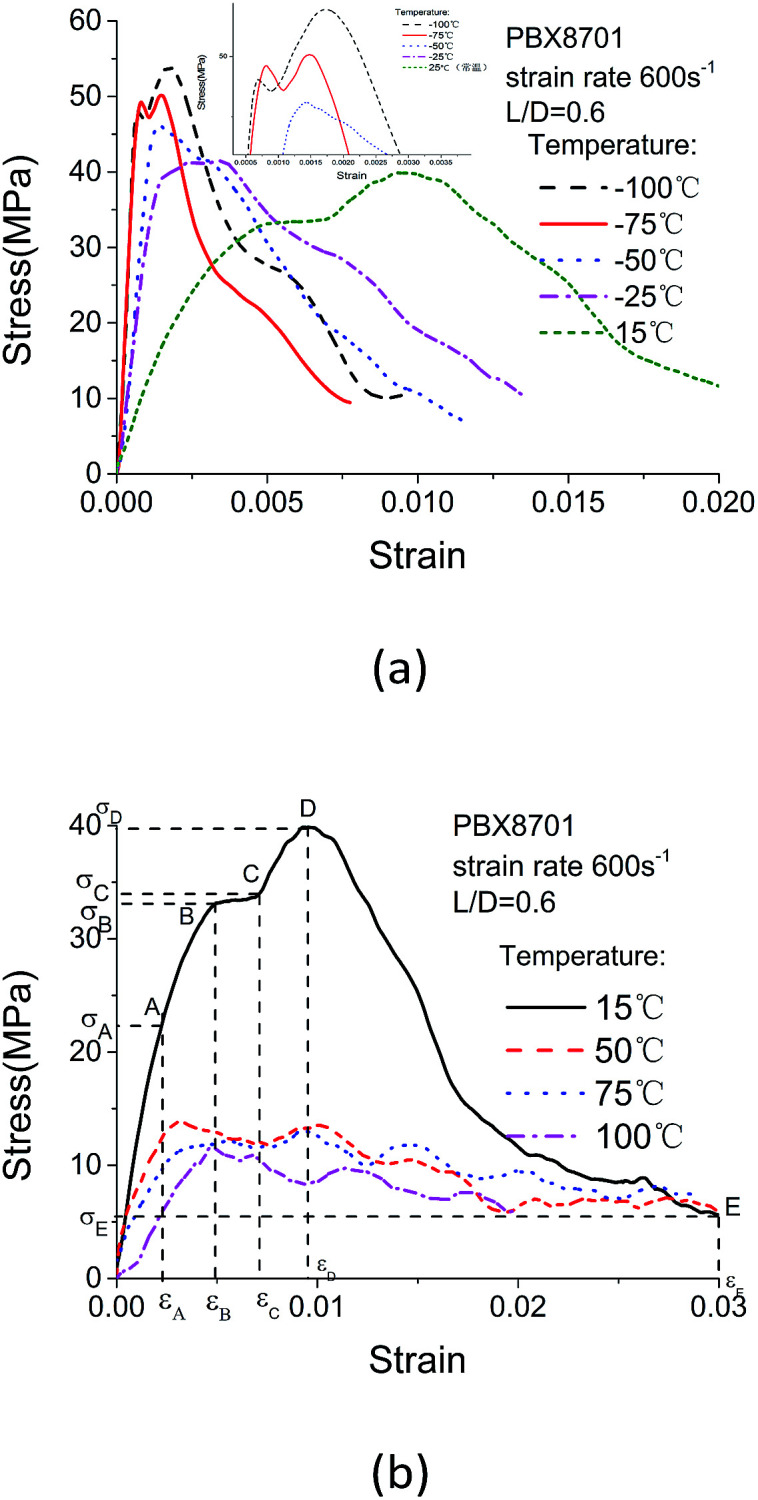
Stress–strain curves obtained at (a) low and (b) high temperatures.

The selected compression curves of 8701 obtained from the high strain-rate test at low and high temperatures are plotted in Fig. 7(a) and (b), respectively.

The development of the stress–strain curves at room temperature is divided into five periods: 0–A, the linear elastic deformation phase; A–B, the tiny plastic deformation phase; B–C, the plastic yield phase (in the repeatability test, the B–C phase does not occur in every experiment); C–D, the mass plastic deformation phase, wherein the material is destroyed at point D; D–E, the material fracture phase, wherein 8701 does not completely lose the ability to resist force. The residual strength enables the material to withstand force and deformation. With decreasing temperature, the plastic phase (A–B, B–C and C–D) decreases, whereas the linear elastic phase (0–A) increases. The deformation of 8701 under decreasing temperature involves the transition from elastic–plastic deformation to elastic–brittle deformation. The stress–strain curves obtained over a range of high temperatures are shown in Fig. 7(b). At high temperatures, the linear elastic phase is relatively small but a long yield process exists. Increasing the temperature from 15 °C to 50 °C causes the stress to decrease rapidly. The stress–strain curves obtained at temperatures exceeding 50 °C are very similar because of the occurrence of binder softening. At this phase, the stress and strain are relatively low.

Drastically different fracture strains are observed in the different temperature regions from −100 °C to 100 °C. The fracture strain increases slightly in the high-temperature regions and is constant under extremely low temperatures. However, the fracture stress increases as the temperature decreases from 100 °C to −100 °C. Wiegand proposed a “constant global-strain to failure” criterion, which is difficult to meet.^21^ Wiegand argues that the damage function is dependent only on strain, and not on temperature/strain rate. However, the failure strain decreases from approximately 1% at 15 °C to 0.2% at −100 °C. The considerable variation in failure strain is most likely due to the contribution of the fracture of organic RDX crystals in 8701.

The stress–strain curves, shown in Fig. 7(a), indicate that serious damage has occurred before the material has completely broken at temperatures of −75 °C and −100 °C in the repeatability test, however, this phenomenon does not occur in every experiment.

**Fig. 8:**
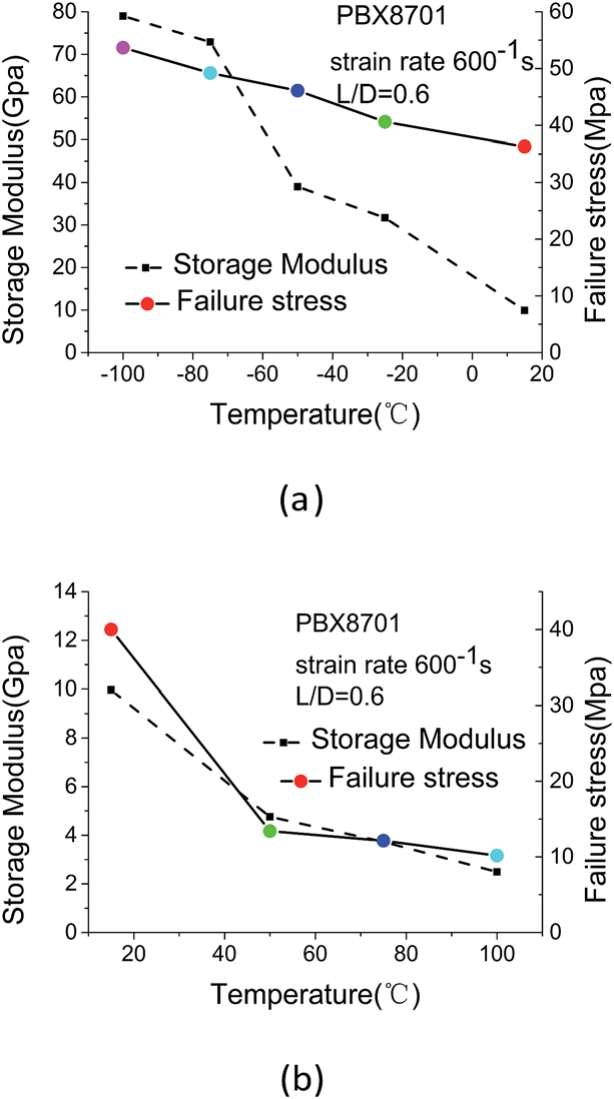
Failure stress and storage modulus data obtained at low and high temperatures.

The failure stress and storage modulus of 8701 at high strain rates are shown in Fig. 8.

The failure stress of 8701 presents a relatively linear dependence on temperature in the temperature ranges of −100 °C to +15 °C and 50–100 °C. However, over the temperature range of 15–50 °C, the temperature dependence of failure stress significantly deviates from a linear pattern during the glass-transition and binder-softening. In contrast to the present results, Gray *et al.* indicated that the failure stresses of PBX9501, XO242-92-4-4, and PBXN-9 are linearly dependent on the test temperature.^16^ Drodge *et al.* and Williamson *et al.* for PBXs are similar to the present results.^19,20^ Establishing the fracture criterion for PBXs at high strain rates is desirable given that fracture stress is a highly reliable parameter.^18–21^ At high strain rates, the storage modulus is negatively correlated with temperature and is linearly dependent on high test temperatures. At temperatures of less than 15 °C, the variation in storage modulus presents an irregular trend.

**Fig. 9:**
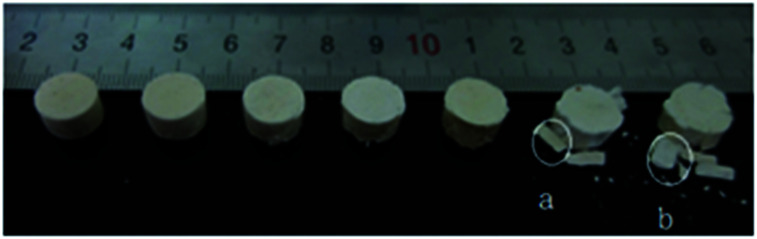
Typical fragments collected at 15 °C (room temperature), 0 °C, −25 °C, −50 °C, −75 °C, −100 °C and −125 °C.

Typical fragments collected from the quasi-static tests performed at low temperatures are shown in Fig. 9. In this figure, the collected sample has been compressed by 12% at a temperature of 15 °C (room temperature), 0 °C, −25 °C, −50 °C, −75 °C, −100 °C, and −125 °C. The results show that cracks have appeared on the surface of the samples at 15 °C and 0 °C. At −25 °C, −50 °C and −75 °C, large cracks and some small fragments appear on the surfaces of the samples. At −100 °C and −125 °C, 8701 has completely broken down starting from its center. 8701 undergoes different crushing processes under the same deformation pressure and different temperatures. Low temperatures are associated with the fast generation and expansion of cracks in 8701.

**Fig. 10:**
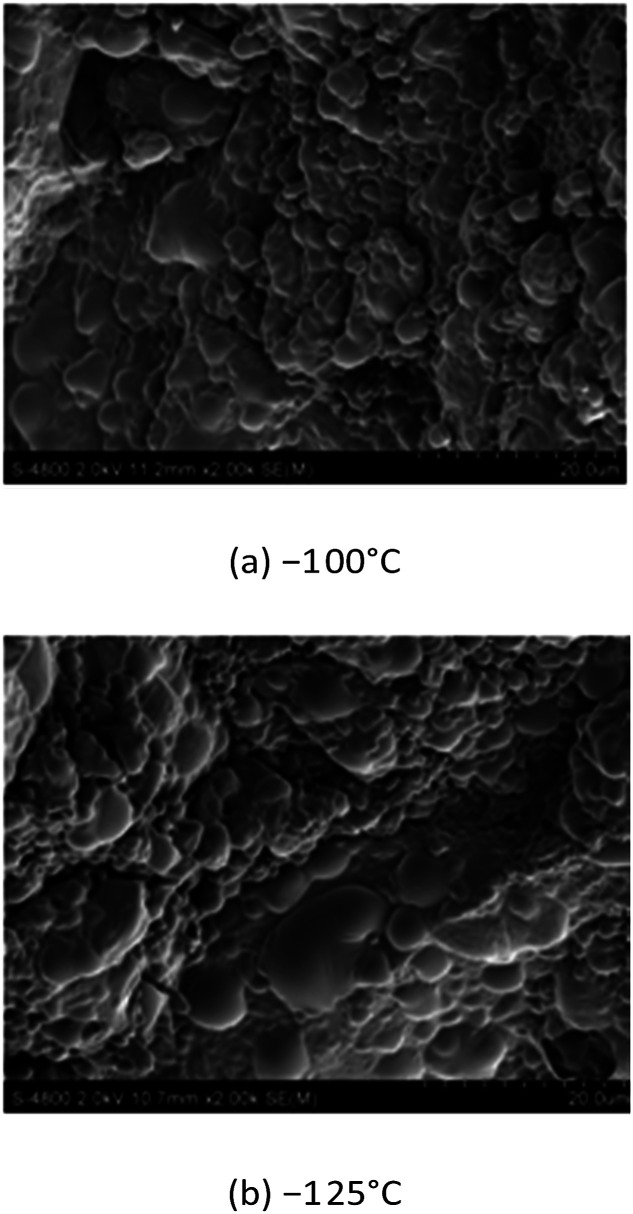
Scanning electron micrograph of 8701 following quasi-static testing at a strain rate of 0.001 s^−1^.

To study the quasi-static fracture mode of 8701 at low temperature, two fragments (a and b in Fig. 9) were taken from the compressed sample at −100 °C and −125 °C. The damage and failure behavior were examined in detail *via* fractographic analysis using SEM. The SEM images are shown in Fig. 10.

Fractured crystals were not observed in Fig. 10. SEM analysis revealed that the failure mode of 8701 at low temperature and under quasi-static loading mainly involves the interface debonding failure of the binder.

**Fig. 11:**
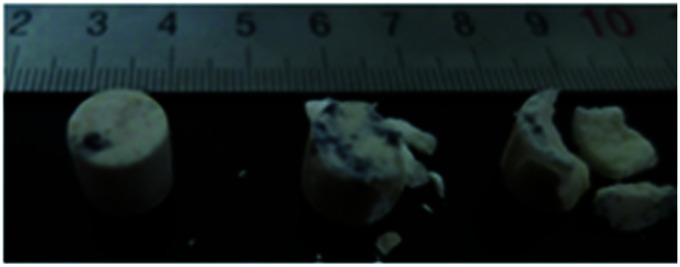
Typical fragments collected after quasi-static tests at 50 °C, 75 °C and 100 °C.

Fig. 11 shows typical fragments subjected to quasi-static testing at high temperature.

The collected samples, shown in Fig. 11, have been compressed by 6% at temperatures of 50 °C, 75 °C and 100 °C. During quasi-static compression at high temperature, bulging easily occurs around the sample as a result of binder softening and melting occurs on the sample surface. At 50 °C, the samples are surrounded by weak bulges and exhibit numerous cracks. At 75 °C, the melting of the sample surface has intensified and fractures have developed on the upper surface of the sample. At 100 °C, the samples have broken into three large fragments.

**Fig. 12:**
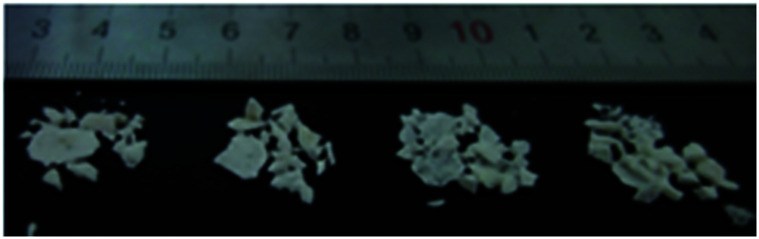
Typical fragments collected after dynamic compression at −25 °C, −50 °C, −75 °C and −100 °C.

Fig. 12 shows typical fragments collected after dynamic compression at low and high temperatures.

Fig. 12 presents images of typical fragments collected after dynamic compression at −25 °C, −50 °C, −75 °C, and −100 °C. The fragmentation of 8701 is strongly dependent on temperature. At low temperature, 8701 breaks into a heap of small fragments as a result of its brittleness. The fragment size decreases as the temperature decreases. This fragmentation pattern indicates that the fracture of 8701 is the dominant mechanical process at low temperature.

**Fig. 13:**
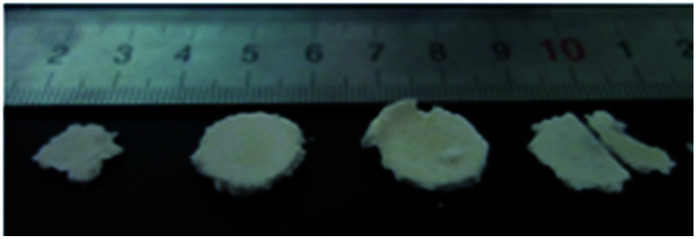
Typical fragments collected after dynamic compression at 15 °C, 50 °C, 75 °C and 100 °C.

Fig. 13 presents images of fragments collected after dynamic compression at 15 °C, 50 °C, 75 °C and 100 °C. The typical fragments obtained at room temperature appear friable and irregularly shaped as a result of the glass transition of 8701. Individual fragments appear spherical because of binder softening at 50 °C. The individual diameters of the fragments are equivalent to that of the Hopkinson bar. The fragments obtained at 100 °C are finer than those obtained at 50 °C. Given that the binder is in a viscous flow state, its ability to resist load is weak.

**Fig. 14:**
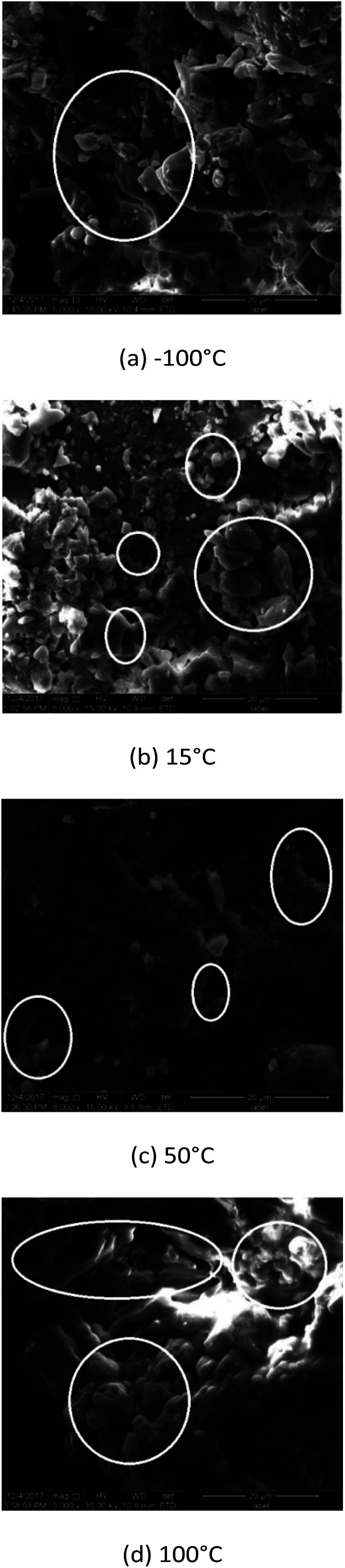
SEM images of 8701 following SHPB testing at a strain rate of 600 s^−1^.

Details of the damage and failure behavior of 8701 specimens at high strain rates were obtained *via* fractographic analysis using SEM. SEM analysis revealed that under a high strain rate and low temperature, 8701 mainly fails due to the transgranular cleavage of RDX crystals. Larger RDX crystal fragments are visible following cleavage fracture (Fig. 14(a)). At a test temperature of 15 °C, the predominant failure mode is the brittle fracture of RDX crystals and the glassy fracture of the binder, as can be seen in Fig. 13(b). Fig. 14(c) and (d) show a completely different phenomenon. No RDX crystals are visible in the images. At 50 °C and 100 °C, the failure mode mainly involves binder softening.

Overall, the main failure mode of 8701 is binder softening, and the RDX organic crystal does not exhibit any obvious damage under quasi-static and dynamic loading at high temperatures. The fracture damage of PBX under quasi-static loading at temperatures below *T*_g_ mainly involves interface debonding inside the binder. Under high-loading rates at temperatures of less than 15.14 °C, the failure of 8701 shows a mixture of interface debonding and transgranular cleavage.

## 4. Discussion

8701 is composed of RDX and polyvinyl acetate whose mechanical properties are controlled by the properties of the two phases and their distributions, as well as the interaction between the two phases. The content of the binder is 2%, but it has a great influence on the mechanical properties of 8701. When the environmental temperature is higher than the glass-transition temperature of polyvinyl acetate (*T*_g_ ≥ 25 °C), it shows very ductile behavior, and when the environmental temperature is lower than the glass-transition temperature of polyvinyl acetate (*T*_g_ < 25 °C), polyvinyl acetate becomes a fragile material.

The fracture stress of solidified polyvinyl acetate is 7.5 MPa at room temperature. The fracture stress of RDX is 35 MPa at room temperature. The fracture stress of 8701 is 9 MPa at room temperature under quasi-static compression, which is closer to the fracture stress of the binder. With decreasing temperature, the fracture strength of polyvinyl acetate can be estimated according to Bourne *et al.*’s research on the mechanical properties of the binder, and it is about 37 MPa at −125 °C. The fracture stress of 8701 is 32 MPa at −125 °C, as was obtained through experiments. The fracture of 8701 is mainly caused by the fracture of the binder under quasi-static compressive loading, which was further demonstrated from the SEM observations, as shown in Fig. 10. As the temperature increases from room temperature to 38 °C, the strength and modulus of 8701 will be greatly reduced due to binder softening. In conclusion, the mechanical properties of the binder have a great influence on 8701 under quasi-static compression. In the process of pressing into a sample, the adhesive force formed between 8701 powders is weaker than that in other positions of the sample. These contact surfaces formed between 8701 powders during pressing may be broken easily in the compressive test.

Under dynamic loading, the modulus of RDX and the binder increases with the decrease of temperature, resulting in the storage modulus of 8701 increasing significantly with the decrease of temperature. The stress–strain curve shows that 8701 has almost no plastic deformation at −50 °C. 8701 became a completely brittle material. At low temperature, 8701 breaks into a heap of small fragments as a result of its brittleness. The typical fragments obtained at room temperature appear friable and irregularly shaped as a result of the glass transition of 8701. Individual fragments appear spherical because of binder softening at high temperature. The mechanical properties of 8701 are mainly determined by polyvinyl acetate and RDX organic crystals, but are considerably influenced by the binder at high temperature.

## 5. Conclusion

The mechanical properties of 8701 were tested over a wide temperature range of −125 °C to 100 °C using the MTS Landmark hydraulic servo test system and SHPB. The stress–strain curves of 8701 exhibit different shapes under quasi-static compression (0.001 s^−1^) and high-rate compression (600 s^−1^) at different temperatures. The binder temperature has a major effect on the mechanical properties of 8701 at temperatures of more than 11.61 °C. The failure stress and storage/elastic modulus of 8701 are negatively correlated with temperature under quasi-static and high strain rates. The fracture mode of 8701 is strongly dependent on temperature. SEM analysis revealed that interface debonding between binders is the predominant failure mode of 8701 under quasi-static loading and at temperatures of less than 15 °C. In contrast, binder softening is the predominant failure mode of PBX8701 under quasi-static loading and at temperatures exceeding 15 °C. The failure mode of 8701 under high-rate loading and at temperatures of less than 15 °C involves a mixture of interface debonding between the binders and transgranular cleavage. The failure mode of PBX8701 under high-rate loading and at temperatures exceeding 15 °C involves binder softening.

## Conflicts of interest

The authors declare no conflict of interest regarding the publication of this paper.

## Supplementary Material
